# Brain Tumor Characterization Using Multiple MR Parameters From Multi‐Contrast EPI With Keyhole (GE‐SE EPIK) Including Oxygen Extraction Fraction: A Comparison to O‐(2‐[18F]Fluoroethyl)‐L‐Tyrosine (FET) Positron Emission Tomography

**DOI:** 10.1002/jmri.29795

**Published:** 2025-04-17

**Authors:** Fabian Küppers, Mohamed Kassem, Seong Dae Yun, Gabriele Stoffels, Christian Filß, Norbert Galldiks, Felix M. Mottaghy, M. Eline Kooi, Karl‐Josef Langen, Philipp Lohmann, N. Jon Shah

**Affiliations:** ^1^ Institute of Neuroscience and Medicine 4 Forschungszentrum Jülich Jülich Germany; ^2^ Cardiovascular Research Institute Maastricht (CARIM) Maastricht University Maastricht the Netherlands; ^3^ Department of Nuclear Medicine RWTH Aachen University Hospital Aachen Germany; ^4^ Department of Neurology Faculty of Medicine and University Hospital Cologne Cologne Germany; ^5^ Institute of Neuroscience and Medicine 3 Forschungszentrum Jülich Jülich Germany; ^6^ Center of Integrated Oncology Aachen Bonn Cologne Duesseldorf (CIO ABCD) Bonn Germany; ^7^ Department of Radiology and Nuclear Medicine Maastricht University Medical Center (MUMC+) Maastricht the Netherlands; ^8^ Institute of Neuroscience and Medicine 11 Forschungszentrum Jülich Jülich Germany; ^9^ JARA—BRAIN—Translational Medicine Aachen Germany; ^10^ Department of Neurology RWTH Aachen University Hospital Aachen Germany

**Keywords:** brain tumor, hybrid MR‐PET, multi‐contrast GE‐SE, multi‐parametric MRI, oxygen extraction fraction

## Abstract

**Background:**

Tumor characterization and treatment efficacy are associated with tissue hypoxia. MR‐derived oxygen extraction fraction (OEF) may offer valuable tumor insights but depends on multiple measurement parameters, often requiring multiple sequence acquisitions. Specific multi‐parametric sequences offer direct access to MR parameter sets within short acquisition times.

**Purpose:**

To evaluate the potential of gradient‐echo spin‐echo echo‐planar imaging with keyhole (GE‐SE EPIK)‐derived parameters (OEF/T_2_/T_2_*/venous cerebral blood volume (vCBV)) to characterize increased metabolic activity tissue identified in [^18^F]fluoroethyl‐L‐tyrosine (FET) PET, serving as a surrogate for neoplastic tissue.

**Study Type:**

Retrospective.

**Population:**

Fifty‐seven brain tumor patients (female/male:31/26; age 27–73 years) with 66 histologically confirmed lesions (suspected glioblastoma (16), glioblastoma (28), astrocytoma (11), metastasis (6), oligodendroglioma (5)).

**Field Strength/Sequence:**

10‐echo GE‐SE EPIK sequence at 3 T.

**Assessment:**

GE‐SE EPIK data were acquired in a hybrid MR PET scanner during FET PET acquisitions. Two tumor segmentations based on FET‐PET uptake and FLAIR hyperintensities were manually created. Mean GE‐SE EPIK‐derived parameters were calculated within tumor regions and compared to contralateral reference values. Relative tumor‐to‐reference parameters were compared across tumor types.

**Statistical Tests:**

One/two‐sampled, two‐tailed *t*‐tests of mean relative MR‐derived parameters. *p*‐value < 0.05 was considered significant.

**Results:**

Significantly increased T_2_/T_2_* and decreased vCBV/OEF were found in FET‐PET and FLAIR‐derived VOIs. Latter showed decreased R2′. Significant correlation between FET uptake and T_2_/T_2_* was found in FET‐VOIs (Pearson correlation: 0.26/0.31, respectively). Oligodendrogliomas showed significant differences to glioblastomas (rR_2_′, rOEF) and astrocytomas (rR_2_′). Metastasis showed different rT_2_ values than suspected gliomas. Astrocytoma differed from gliomas in FET‐TBR. Susceptibility artifacts in T_2_* maps from air‐tissue interfaces limited qualitative data interpretation.

**Data Conclusion:**

GE‐SE EPIK provides multiple MR parameters that are sensitive to expected changes in tumor regions obtained from FET and FLAIR thresholds. Susceptibility artifacts in T_2_*/OEF maps made the differentiation between tumor relapse and treatment‐related changes challenging. However, certain MR‐derived parameters showed the ability to distinguish tumor types.

**Evidence Level:**

3.

**Technical Efficacy:**

Stage 2.

**Plain Language Summary**

- A novel and fast MRI sequence (GE‐SE EPIK) was used to assess brain tumors by measuring parameters such as the oxygen extraction fraction (OEF) and other tissue properties within a 2‐min measurement time.
- These MRI findings were compared with tumor volumes derived from FET‐PET imaging and standard MRI techniques.
- Results showed significant differences between the tumor volumes and healthy tissue.
- However, while some MRI parameters correlated with PET tracer uptake, OEF measurements did not.
- Differences between tumor types were minimal, while overall imaging artifacts and treatment‐related changes limited the approach's robustness.
- Future work is required to strengthen its clinical relevance.

## 1 Introduction

Oxygen is the most fundamental and essential element in cellular metabolism. Numerous enzymes, including oxygenase, rely on oxygen for their functioning, and oxidative phosphorylation generates a greater amount of energy compared to glycolysis [1]. The absence of oxygen in tissue results in hypoxia, a feature of numerous cancers, which is associated with the growth of tumors and poorer clinical outcomes [2]. Of all the organs in the body, the brain demands the greatest quantity of oxygen. Even though the brain constitutes just 2% of the total body weight, it utilizes 20% of the body's oxygen supply [3]. Oxygen consumption mainly depends on extracting oxygen from arterial blood, and the oxygen extraction fraction (OEF) has been proposed as a potential biomarker for detecting hypoxia [4]. In other words, when tissue does not receive an adequate supply of oxygen (hypoxia), it responds by showing a decrease in OEF [5, 6, 7]. A preclinical study in glioma‐bearing rats has demonstrated that intra‐tumoral hypoxic regions showed decreased OEF as measured by ^18^F‐fluoromisonidazole (FMISO) PET [8]. A direct comparison in high‐grade gliomas between severe tissue hypoxia measured by FMISO and vascular deoxygenation, as characterized by relative oxygen extraction fraction (rOEF) from subsequent T_2_ and $T_{2}^{*}$ measurements, has shown poor spatial correspondence [9]. Nevertheless, the association of hypoxia with higher tumor grade is commonly accepted [10, 11], and the importance of hypoxia to therapy resistance has been reported in several studies [12, 13].

However, altered OEF is not specific to hypoxia; it can also occur in other conditions where oxygen delivery is compromised, such as in ischemia (reduced blood flow) [14] or anemia (reduced oxygen‐carrying capacity of blood) [15]. The assessment of OEF in humans has traditionally relied on PET with ^15^O‐labeled radiotracers [16]. In parallel, 2‐nitroimidazole compounds like FMISO have been employed for the detection of hypoxic tumors, including gliomas, and FMISO is now the most widely used PET imaging agent for assessing hypoxia [17, 18, 19]. While ^15^O‐PET remains the reference standard for OEF mapping [20], its widespread clinical application has been hindered by several factors, including logistical complexities, radiation exposure, and the necessity for an on‐site cyclotron to generate the short‐lived ^15^O isotope, which has a physical half‐life of approximately 2 min [21]. A further drawback associated with FMISO PET imaging is the low signal‐to‐noise ratio (SNR), which may impact the accuracy and reproducibility of the imaging results [22].

**FIGURE 1:**
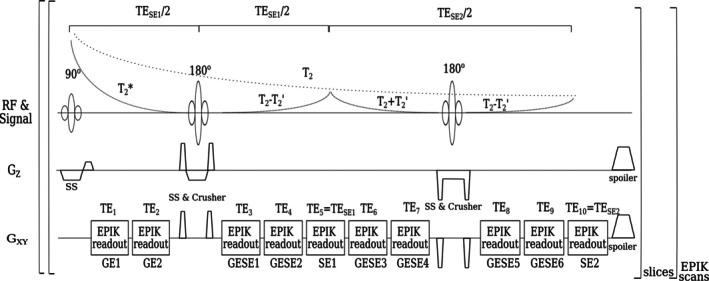
Sequence diagram of the 10‐echo GE‐SE EPIK sequence along with the theoretical signal decay from the combined contrasts.

MRI techniques can be employed to assess tumor perfusion, offering indirect insights into the presence of hypoxia and angiogenesis within malignant tissues. Blood‐oxygen‐level‐dependent (BOLD) MRI [23, 24], which is also referred to as intrinsic susceptibility‐weighted MRI, is a non‐invasive technique that indirectly assesses alterations in tissue oxygenation. The growing interest in the use of MR techniques to quantify OEF has driven the development of the gradient‐echo (GE) spin‐echo (SE) sequence based on echo planar imaging (EPI) with keyhole readouts, known as GE‐SE EPIK [25]. This sequence acquires 10 echoes, of which 2 are pure GE, 7 are mixed GE‐SE, and 2 are pure SE, as shown in the sequence diagram in Figure 1. This sequence thereby provides a rapid and contrast agent‐free approach to simultaneously measure T_2_ and $T_{2}^{*}$. These transverse relaxation properties are both related to blood oxygenation and, hence, enable the quantification of OEF. In an earlier study, the benefits of the GE‐SE EPIK sequence when compared to EPI‐based multi‐echo multi‐contrast methods were demonstrated [19]. These advantages include improved spatial resolution, superior temporal resolution, and the acquisition of a larger number of echoes, including two purely SE. This previous study demonstrated the precision of simultaneous T_2_ and $T_{2}^{*}$ measurements in healthy individuals compared to reference techniques and also demonstrated the sensitivity of OEF measurements during breath‐hold experiments. The general advantages of a simultaneous acquisition scheme compared to sequential measurements, as performed, for example, by Preibisch et al. [9], are a shorter measurement time, better temporal resolution, and intrinsically registered parameter maps from the same physiological state.

In addition to structural MRI, amino acid PET imaging using the tracer O‐(2‐[^18^F]fluoroethyl)‐L‐tyrosine (FET) is also a widely accepted diagnostic method for patients with brain tumors and can be used to inform, for example, the differentiation between tumor relapse and treatment‐related changes, metabolic tumor volume for planning of surgery or radiotherapy, and treatment monitoring or biopsy guidance [26, 27, 28, 29]. Furthermore, an increased uptake of FET, which indicates an increase in metabolic activity, is associated with an unfavorable outcome (i.e., shorter progression‐free and overall survival) as well as a reduced response to therapy [30, 31].

Thus, the aim of this study was to investigate the association between FET PET and the multi‐parametric output from the GE‐SE EPIK sequence (T_2_, $T_{2}^{*}$, their inverse difference ($R_{2}^{\prime }$), venous cerebral blood volume (vCBV), and OEF). Moreover, this study aimed to investigate whether these MR‐derived quantitative measures could provide complementary information that may help in the differentiation of treatment‐related changes from tumor relapse.

## 2 Patients and Methods

### 2.1 Patient Cohort

The local institutional review board approved the study protocols, screening questionnaires, and consent forms used in this study. Ethical approval (096/18) was provided by the local ethics committee. Written informed consent was obtained from all patients prior to scanning. Assessment criteria led to the exclusion of patients with FET‐TBR values below 1.6 or with tumor volumes below 0.5 mL as they were defined as non‐measurable disease according to [32]. Sixty‐six data sets were acquired from 57 patients with brain tumors (31 females and 26 males with a median age of 52 and a range of 27 to 73 years). Subgroups according to different tumor types were defined as follows: suspected glioblastoma where no tissue samples were available for classification (16), glioblastoma (28), astrocytoma (11), metastasis (6), and oligodendroglioma (5). While 21 patients were untreated, the majority of the cohort had undergone various treatments, differing in type, number, and combination, prior to the MR‐PET acquisition. Among these, surgery, radiotherapy, and chemotherapy were the most common, while a small number of patients had received radiosurgery, tumor treating fields therapy, or immunotherapy. Further details on the individual treatment history and the tumor histology of the patients are provided in Table 1.

**TABLE 1:** Characteristics of the patient cohort, including sex, age, tumor histology, and treatment history.

		Quantity	Range/Percentage
Sex (male/female)		31/26	(54%/46%)
Age (years)		52	(27–73)
WHO CNS 2021 Tumor Type			
GBM, IDH wildtype		28	(42%)
Suspected Glioma^a^		16	24%
Astrocytoma, IDH‐mutated, Grade (2,3,4)		6/4/1	17%
Metastasis		6	9%)
Oligodendroglioma, IDH‐mutated, 1p19q codeleted, Grade (2,3)		2/3	8%
Lesion Volumes (mL):			
PET‐SUV		16.6	0.5–79.7
FLAIR		53.5	1.4–258.0
Treatment (Number of Procedures)			
Surgery	(1/2/3)	28/8/3	
Radiotherapy	(1/2)	31/6	
Chemotherapy	(1)	32	
Radiosurgery	(1/3)	3/2	
Tumor treating fields		3	
Immunotherapy		4	
Untreated		21	

^a^ No tissue samples available for classification.

### 2.2 MRI Acquisition

MR data were acquired on a 3 T hybrid MR/PET scanner (TRIO; Siemens, Erlangen, Germany) with 40 mT/m gradient strength and 200 T/m/s gradient slew rate. A 20‐channel transmit coil was used for data acquisition. An anatomical MPRAGE sequence was acquired with 256 slices, repeat time (TR) of 2000 ms, echo time (TE) of 3.03 ms, 176 × 240 matrix size with 1 × 1 × 1 mm^3^ resolution, and flip angle (FA) of 9°. In addition, a T2‐weighted Fluid‐attenuated inversion recovery (FLAIR) sequence with 144 slices, TR of 6000 ms, TE of 403 ms, 256 × 240 matrix size with 0.97 × 0.97 × 2 mm^3^ resolution, and flip angle (FA) of 120° was acquired. This was followed by a 10‐echo GE‐SE EPIK sequence [25] acquisition with the following imaging parameters: 44 slices, TR of 6900 ms, TE = 10,20,41,51,66,76,86,107,117,132 ms, 128 × 128 matrix size, 1.9 × 1.9 × 3 mm^3^ resolution, and FA of 90°. Two‐fold GRAPPA acceleration with EPI‐based reference kernels, navigator‐based phase correction, and a SPARSE EPIK factor of 14 were implemented to allow sufficiently short TEs for all echoes [25]. After data recombination based on the EPIK acquisition scheme, phase correction, and GRAPPA reconstruction were performed prior to the final Fourier transformation using an in‐house developed reconstruction script. GE‐SE EPIK data allowed simultaneous quantification of relaxometry parameters and oxygen extraction fraction information in 2:03 min acquisition time.

### 2.3 PET Acquisition

The amino acid tracer FET was produced via nucleophilic ^18^F‐fluorination with a radiochemical purity > 98%, molar radioactivity > 200 GBq/μmol, and a radiochemical yield of approximately 60% [33]. According to international guidelines for brain tumor imaging using radiolabeled amino acid analogs [34], patients fasted for at least 4 h before the PET measurements. All patients underwent a dynamic PET scan from 0 to 50 min after injection of 3 MBq of FET per kg of body weight. PET imaging was performed simultaneously with 3 T MR imaging using a BrainPET insert (Siemens, Erlangen, Germany; axial field of view, 19.2 cm). The BrainPET is a compact cylinder that fits into the bore of the Magnetom Trio MR scanner [35]. Iterative reconstruction parameters were two subsets and 32 iterations using the OPOSEM algorithm [36] for the BrainPET. Data were corrected for random, scattered coincidences, dead time, and motion. Attenuation correction was based on a template‐based approach. The reconstructed dynamic data sets consisted of 16 time frames (5 × 1 min, 5 × 3 min, 6 × 5 min).

### 2.4 MRI Data Processing

The signal evolution of the 10‐echo GE‐SE EPIK data was fitted using a nonlinear least squares algorithm to provide voxel‐wise T_2_ and $T_{2}^{*}$ relaxation time maps. The underlying theoretical signal equation for the fitting procedure is given by.

(1) $$S\left(t\right)=\left\{\begin{array}{c} S_{0}\times e^{-t\times R_{2}^{*}}\;\text{for}\;0<t<{\mathrm{TE}}_{\mathrm{SE}1}/2\;\\ \frac{S_{0}}{\delta }\times e^{-{\mathrm{TE}}_{\mathrm{SE}1}\left(R_{2}^{*}-R_{2}\right)}\times e^{-t\left(2\times R_{2}-R_{2}^{*}\right)\;}\;\text{for}\;{\mathrm{TE}}_{\mathrm{SE}1}/2<t⩽{\mathrm{TE}}_{\mathrm{SE}1}\;\\ \frac{S_{0}}{\delta }\times e^{+{\mathrm{TE}}_{\mathrm{SE}1}\left(R_{2}^{*}-R_{2}\right)}\times e^{-tR_{2}^{*}}\;\text{for}\;{\mathrm{TE}}_{\mathrm{SE}1}<t<\frac{1}{2}\times \left({\mathrm{TE}}_{\mathrm{SE}1}+{\mathrm{TE}}_{\mathrm{SE}2}\right)\;\\ \frac{S_{0}}{\Delta }\times e^{-{\mathrm{TE}}_{\mathrm{SE}2}\left(R_{2}^{*}-R_{2}\right)}\times e^{-t\left(2\times R_{2}-R_{2}^{*}\right)\;}\;\text{for}\;\frac{1}{2}\times \left({\mathrm{TE}}_{\mathrm{SE}1}+{\mathrm{TE}}_{\mathrm{SE}2}\right)<t⩽{\mathrm{TE}}_{\mathrm{SE}2} \end{array}\right.$$

Here, *S*_0_ is the net magnetization, and Δ and δ are correction factors that consider slice profile mismatches as well as pulse imperfections [37] between the excitation and refocusing pulses within the GE‐SE EPIK sequence. Based on the two transverse relaxation times, $R_{2}^{\prime }$ is computed by

(2) $$R_{2}^{\prime }=\frac{1}{T_{2}^{\prime }}=\frac{1}{T_{2}^{*}}-\frac{1}{T_{2}}$$

The venous cerebral blood volume (vCBV) is defined as a fraction of the voxel volume and therefore dimensionless. It is obtained with the following equation [38]:

(3) $$\text{vCBV}=\frac{S_{\text{extr}}}{S\left({\mathrm{TE}}_{\mathrm{SE}}\right)}$$

where *S*_extr_ is the extrapolated signal at the spin echo time based on the neighboring mixed GESE echoes (echoes 3, 4, 6, and 7), and *S*(TE_SE_) is the signal directly acquired at the SE (5th echo).

Finally, the OEF is given by the qBOLD theory [39].

(4) $$\mathrm{OEF}=\frac{{R_{2}}^{\prime }}{\text{vCBV}\times 4/3\times \pi \times \gamma \times \Delta \chi_{0}\times \mathrm{Hct}\times B_{0}}$$

Here, the input T_2_ and $T_{2}^{*}$ maps are spatially smoothed by a Gaussian kernel with a width of 3 mm before calculating $R_{2}^{\prime }$, and an oxygen saturation close to 100% is assumed. The magnetic field B_0_ is 3 T and the gyromagnetic ratio is γ = 2.68 rad·s^−1^·T^−1^, the fractional hematocrit Hct is 0.36 [40, 41], and the susceptibility difference between the fully oxygenated and deoxygenated blood is given by Δχ_0_ given by 0.246 ppm per unit Hct [42].

### 2.5 PET Data Processing

Summed PET images obtained between 20 and 40 min following injection were selected for the analysis. Tumor segmentation was performed by an experienced neurosurgeon with 3 years of experience (MK) with a threshold of 1.6 using a 3D auto‐contouring process from the PMOD software (version 4.2; PMOD Technologies LLC, Faellanden, Switzerland). Tumor segmentations were checked by an experienced nuclear medicine physician with more than 30 years of experience (KJL). This threshold is based on a biopsy‐controlled study in patients with gliomas and was found to differentiate best between tumoral and peritumoral tissue [43]. It further follows clinical recommendations [34]. Mean Tumor‐Background Ratios, TBRs (TBR_mean_) were calculated by dividing the mean standardized uptake values (SUV) of the tumor segmentation by the mean SUV of larger regions of interest (ROIs) placed in the centrum semiovale of the contralateral unaffected hemisphere and included white and gray matter.

### 2.6 Tumor Volume Analysis

To compare FET‐PET and MR parameters, both datasets, as well as FLAIR images, were coregistered to the higher‐resolution anatomical MP‐RAGE data by using the SimpleITK (https://simpleitk.org/) and Scipy (https://scipy.org/) python toolboxes. GE‐SE EPIK images were resampled to match the MP‐RAGE image spacing, size, and orientation before coregistration. The resulting affine transformation matrix was then applied to the MR parameter maps. Hence, the T_2_, $T_{2}^{*}$, vCBV, and OEF maps had the same voxel size as MP‐RAGE and were coregistered with the FET images.

The previously segmented FET PET tumor volumes‐of‐interest (VOIs), as well as VOIs derived from thresholded hyperintense regions in the FLAIR images, were applied to the T_2_, $T_{2}^{*}$, $R_{2}^{\prime }$, vCBV, and OEF MR parameter maps, and the mean tumor values were calculated for both VOI types. The mean values of reference tissue in the corresponding contralateral unaffected hemisphere were also calculated. Relative measures of each MR parameter were obtained by dividing the mean tumor values by the reference values, to provide a direct indication of whether the tumor values were elevated (> 1) or reduced (< 1) compared to contralateral healthy brain tissue. For example, relative OEF values, rOEF, were computed as follows:

(5) rOEF=OEF¯tumourOEF¯contralateral

### 2.7 Statistical Analysis

One‐sampled, two‐tailed *t*‐tests were used to determine whether the mean relative measures of each MR‐derived parameter were significantly > 1 or < 1. All tumor subgroup combinations, without correction for multiple testing, were analyzed for each MR‐derived parameter using a two‐sample, two‐tailed *t*‐test to evaluate whether the MR parameters differed significantly between tumor types. A *p* value < 0.05 was considered significant.

## 3 Results

**FIGURE 2:**
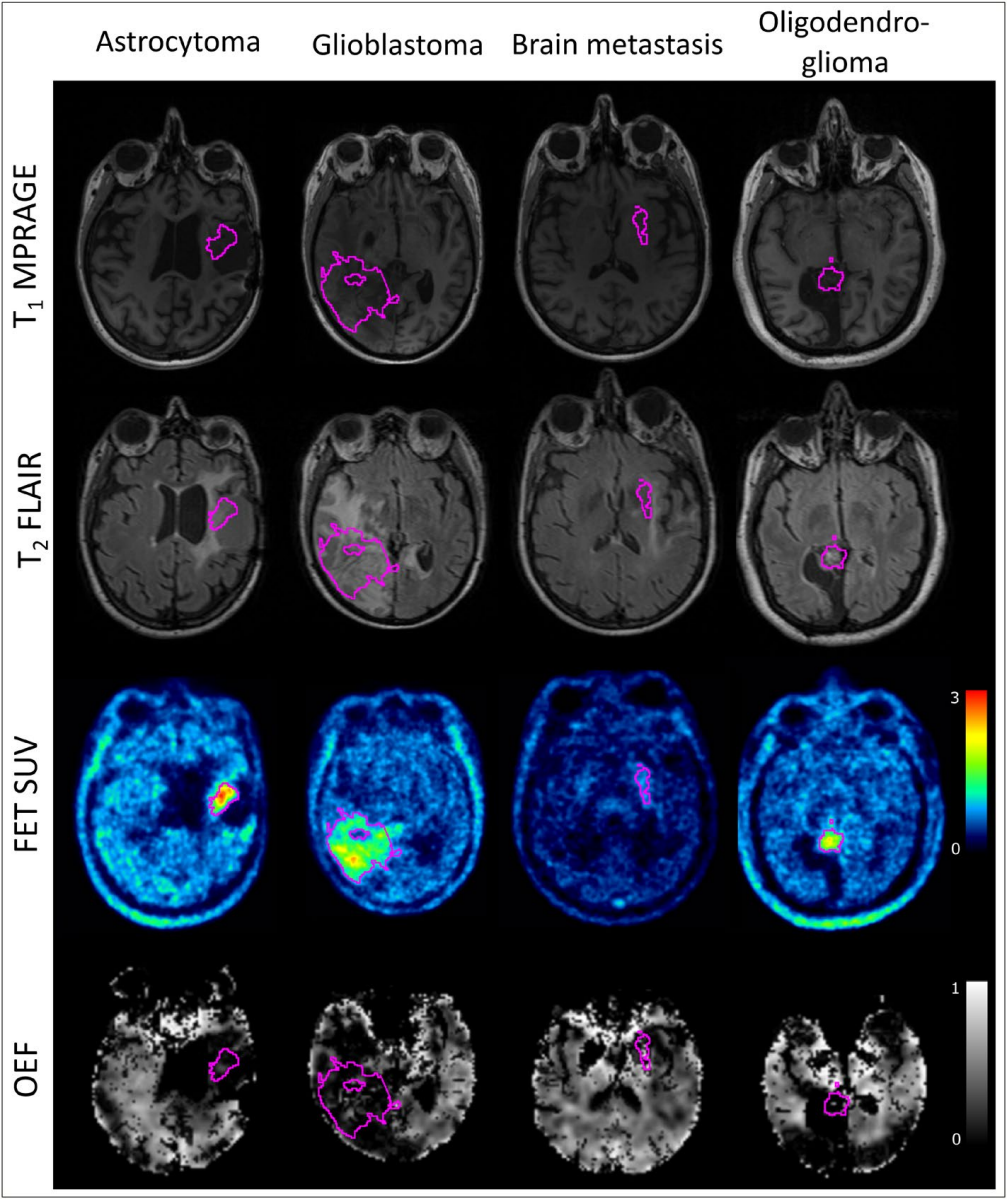
Representative images of the anatomical T1 MP‐RAGE scan (top), T2w FLAIR images (2nd row), the FET SUV map (3rd row) and OEF maps (bottom) for different tumor types, that is, astrocytoma, glioblastoma, metastasis, and oligodendroglioma, from left to right. Tumor VOIs derived from FET PET thresholds are overlayed with pink outlines.

**FIGURE 3:**
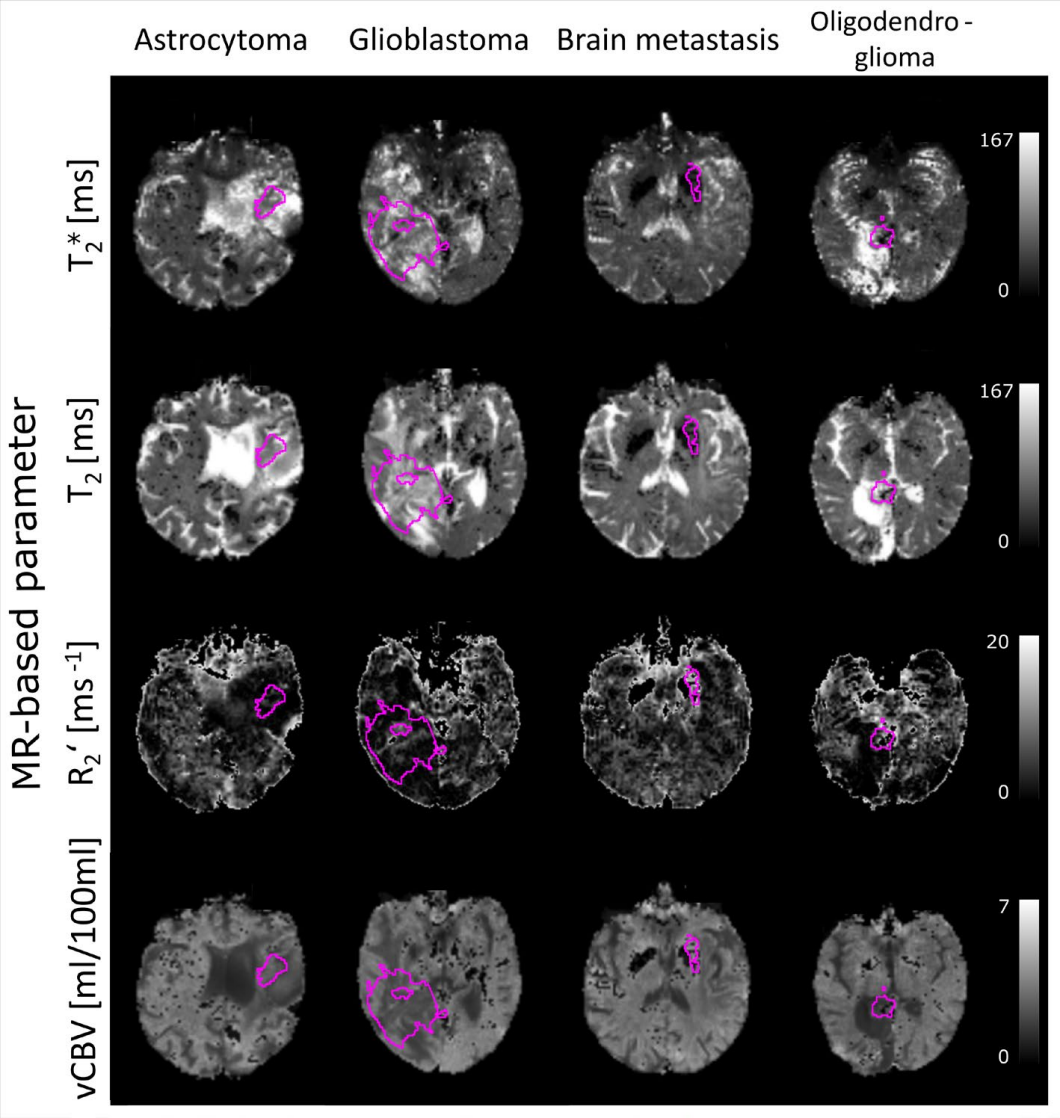
Representative images of the quantitative parameter maps from the 10‐echo GE‐SE EPIK sequence. From top to bottom, $T_{2}^{*}$, T_2_, $R_{2}^{\prime }$ and vCBV are shown for different tumor types, that is, astrocytoma, glioblastoma, metastasis, and oligodendroglioma, from left to right. Tumor VOIs derived from FET PET thresholds are overlayed with pink outlines.

A comparison of the T1‐weighted MP‐RAGE and the T2‐weighted FLAIR scans, along with the FET SUV and OEF maps from GE‐SE EPIK are shown in Figure 2. Representative slices from four patients with different tumor types are shown. MR‐parameter maps (T_2_, $T_{2}^{*}$, $R_{2}^{\prime }$, and vCBV) for the same selected patients and slices are presented in Figure 3 where it can be seen that the MR parameter maps include larger areas of altered contrasts compared to the tumor VOI derived from FET uptake. These additional areas closer match the VOIs derived from FLAIR contrasts, as shown in Figures S1 and S2.

**FIGURE 4:**
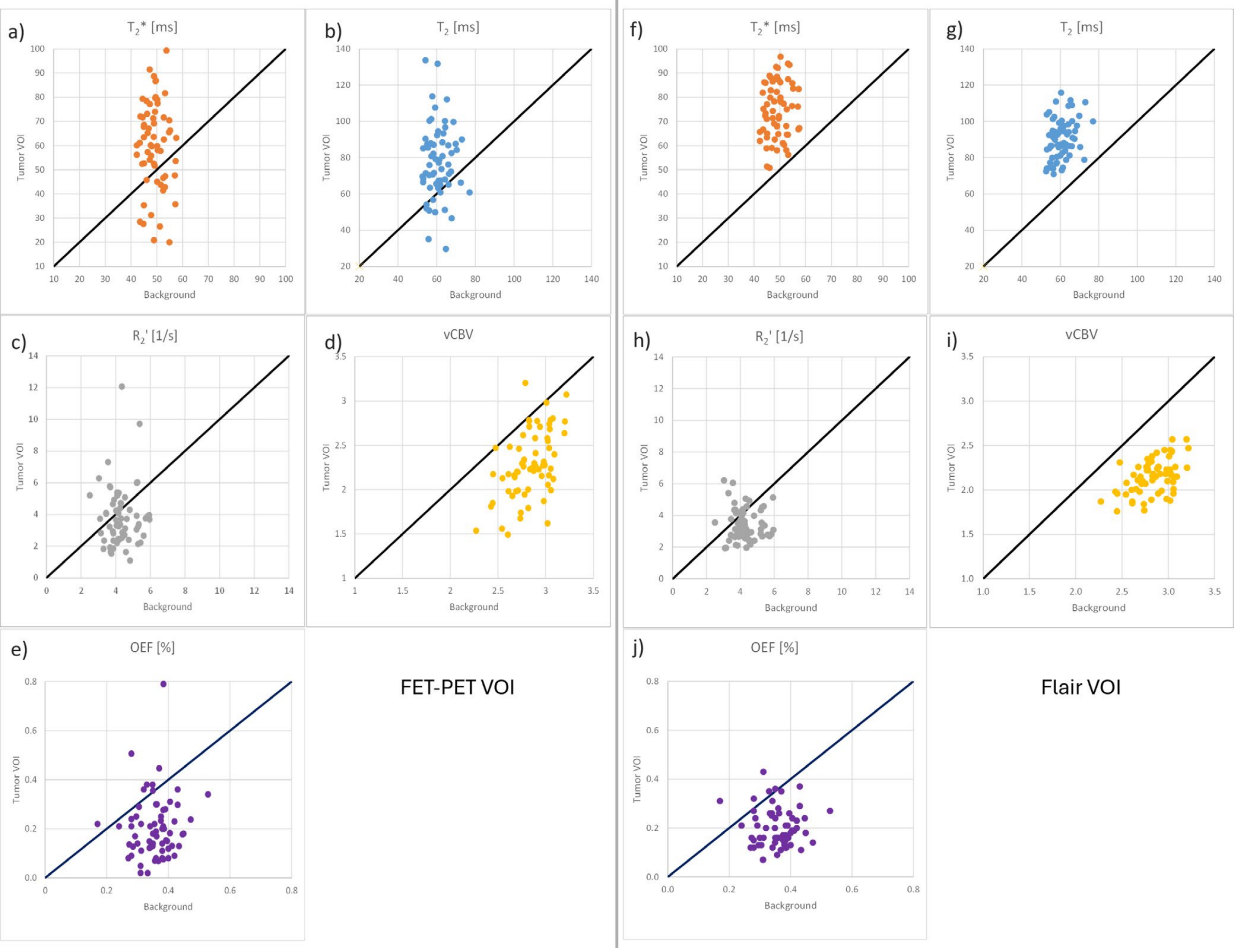
Scatter graphs of quantitative MR parameters (a,f: $T_{2}^{*}$, b,g: T_2_, c,h: $R_{2}^{\prime }$, d,i: VCBV and e,j: OEF) comparing the mean values within the FET‐derived tumor volumes (a–e) as well as FLAIR‐derived volumes (f–j) and the reference contralateral background VOI. Identity lines are shown in black.

**FIGURE 5:**
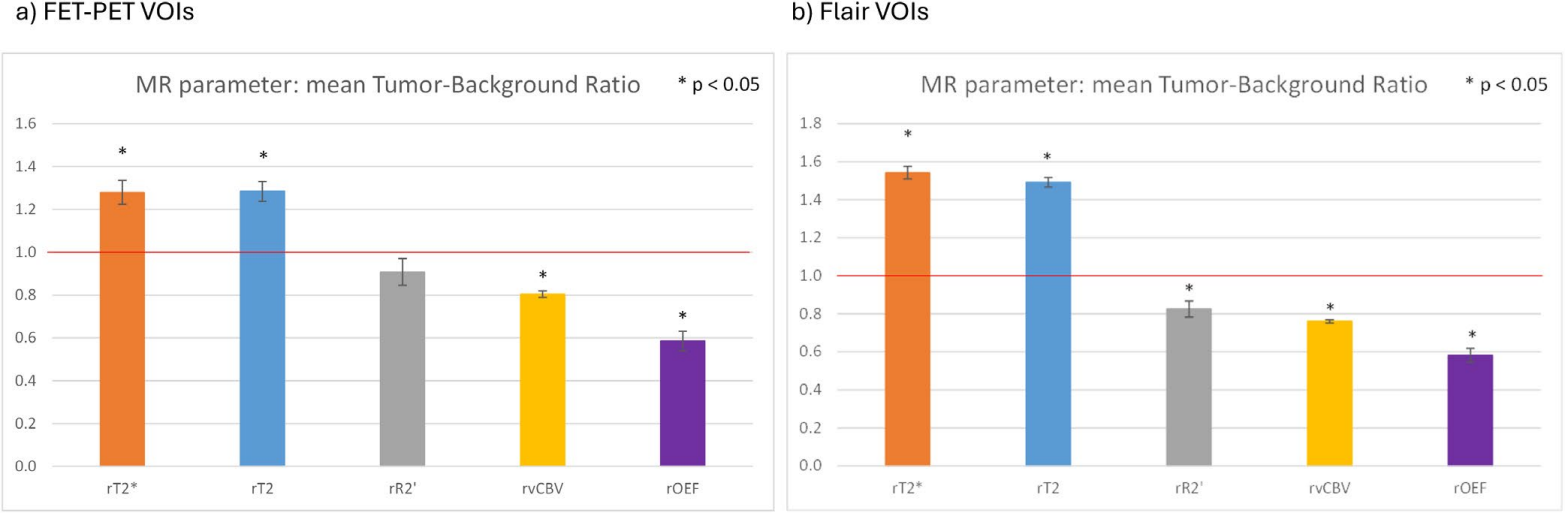
Summary of the mean values of the relative MR parameter measures in FET‐PET (a) and FLAIR (b) derived VOIs (tumor_VOI/reference) including the standard error of the mean (SEM). The red horizontal line marks the equality of tumor values to reference contralateral values. Significant differences with *p*‐values below 0.05 are obtained for OEF, T2, $T_{2}^{*}$, and vCBV in both VOI sets, while the mean ${\mathrm{rR}}_{2}^{\prime }$ shows only significant differences in FLAIR VOIs.

Figure 4 and Table 2 show the mean MR parameters within the tumor VOIs for each patient compared to the corresponding reference contralateral values in the form of scatter plots. The average healthy tissue values in the contralateral hemisphere for the whole patient cohort were: T_2_ 60.8 ± 5.4 ms, $T_{2}^{*}$ 49.2 ± 3.9 ms, $R_{2}^{\prime }$ 4.30 ± 0.75 s^−1^, vCBV 2.84 ± 0.20 and OEF 0.36 ± 0.06. Compared to the reference contralateral values, T_2_ and $T_{2}^{*}$ were generally increased in both tumor VOIs from FET‐PET and FLAIR, whereas vCBV and OEF were generally decreased. Mean $R_{2}^{\prime }$ values were only slightly reduced in FET‐VOIs with a *p*‐value of 0.07. Statistical comparisons are shown in Figure 5, where it can be seen that rT_2_ and ${\mathrm{rT}}_{2}^{*}$ were significantly higher than one (1.28 ± 0.05 and 1.88 ± 0.06 in FET VOIS, respectively; 1.49 ± 0.03 and 1.54 ± 0.03 in FLAIR VOIs, respectively), rvCBV and rOEF values were significantly lower than one (0.80 ± 0.02 and 0.59 ± 0.05 in FET VOIs, respectively; 0.76 ± 0.02 and 0.58 ± 0.04 in FLAIR VOIs, respectively), and ${\mathrm{rR}}_{2}^{\prime }$ was 0.91 ± 0.06 in FET VOIs and 0.83 ± 0.04 in FLAIR VOIs. The mean TBR was 2.10 ± 0.04.

**TABLE 2:** Overview of the tumor volume, TBR, and relative MR parameters within the PET‐SUV defined tumor volume for all acquired patients.

#No	Histology	FET VOI							FLAIR VOI					
		Volume	rT_2_	rT_2_*	rR_2_’	rvCBV	rOEF	TBR	Volume	rT_2_	rT_2_*	rR_2_’	rvCBV	rOEF
1	Suspected glioma	39.42	1.35	1.43	0.61	0.81	0.62	2.52	46.90	1.32	1.26	0.80	0.90	0.91
2	Suspected glioma	0.77	0.69	0.36	0.48	0.81	0.47	1.74	0.00	X	X	X	X	X
3	Suspected glioma	0.93	1.32	0.99	2.07	0.83	0.71	1.72	3.23	1.38	1.10	0.80	2.05	1.38
4	Suspected glioma	2.45	1.04	0.89	1.58	0.99	1.02	1.83	57.04	1.20	1.11	0.84	1.31	1.03
5	Suspected glioma	3.14	1.25	1.23	1.05	0.73	0.52	1.95	48.85	1.81	1.90	0.64	0.79	0.31
6	Suspected glioma	9.20	1.54	1.75	0.48	0.78	0.20	2.01	24.01	1.60	1.76	0.75	0.57	0.25
7	Suspected glioma	1.10	2.18	1.54	1.55	0.61	0.16	1.80	36.34	1.92	1.92	0.67	0.85	0.23
8	Suspected glioma	39.15	1.97	1.81	0.73	0.63	0.37	2.12	75.67	1.92	1.81	0.64	0.73	0.42
9	Suspected glioma	8.75	1.72	1.53	0.91	0.64	0.29	1.86	42.17	1.71	1.64	0.65	0.93	0.37
10	Suspected glioma	1.08	1.22	1.17	0.98	0.89	0.83	2.02	6.13	1.49	1.44	0.78	0.76	0.78
11	Suspected glioma	24.40	1.45	2.08	0.36	0.74	0.21	2.77	34.17	1.42	1.77	0.74	0.47	0.34
12	Suspected glioma	1.19	1.56	1.85	0.54	0.76	0.32	1.92	104.94	1.69	1.74	0.71	0.72	0.43
13	Suspected glioma	18.47	1.20	1.10	1.03	0.81	1.15	2.41	13.42	1.22	1.17	0.79	0.85	1.06
14	Suspected glioma	5.68	2.47	2.36	0.59	0.54	0.20	2.13	70.38	1.94	1.96	0.62	0.79	0.45
15	Suspected glioma	1.63	1.79	1.61	1.17	0.73	0.88	1.87	65.92	1.62	1.44	0.76	1.09	0.88
16	Suspected glioma	4.00	1.06	1.50	0.51	0.92	0.22	2.30	0.00	X	X	X	X	X
17	Astrozytom WHO II	0.62	1.61	1.78	0.82	0.79	0.37	1.68	11.17	1.96	1.93	0.68	1.05	0.40
18	Astrozytom WHO II	0.50	1.29	0.89	0.87	0.84	1.21	1.83	1.39	1.22	1.05	0.85	1.17	0.95
19	Astrozytom WHO II	0.76	0.63	0.62	0.55	0.78	0.40	1.74	7.23	1.43	1.31	0.70	1.64	0.54
20	Astrozytom WHO II	2.90	1.23	2.15	0.23	0.76	0.06	2.02	27.26	1.52	1.97	0.72	0.50	0.23
21	Astrozytom WHO II	0.50	0.95	0.65	0.41	0.73	0.21	1.58	57.70	1.41	1.53	0.83	0.62	0.48
22	Astrozytom WHO II	49.21	1.18	1.18	0.89	0.77	0.75	2.44	39.02	1.34	1.22	0.73	1.19	0.96
23	Astrozytom WHO III	6.50	1.32	1.42	0.75	0.85	0.72	2.07	11.03	1.57	1.55	0.74	0.83	0.54
24	Astrozytom WHO III	4.80	1.41	1.63	0.47	0.80	0.32	2.23	37.52	1.39	1.57	0.84	0.50	0.46
25	Astrozytom WHO III	0.62	1.82	1.17	1.81	0.70	0.64	1.82	51.05	1.45	1.44	0.80	0.85	0.51
26	Astrozytom WHO III	5.17	1.26	1.33	0.63	0.90	0.69	1.67	8.44	1.40	1.46	0.80	0.64	0.67
27	Astrozytom WHO IV	6.80	1.22	1.41	0.44	0.84	0.36	2.03	19.41	1.33	1.19	0.81	1.18	0.77
28	Brain metastasis	15.00	1.09	1.19	0.76	0.96	0.72	2.18	68.02	1.53	1.57	0.76	0.75	0.44
29	Brain metastasis	22.32	0.98	0.90	1.21	0.98	0.55	2.09	61.01	1.57	1.72	0.75	0.55	0.31
30	Brain metastasis	1.15	0.98	1.15	0.58	0.99	0.39	1.77	15.93	1.33	1.51	0.79	0.45	0.41
31	Brain metastasis	1.90	0.46	0.43	1.15	0.64	0.55	1.94	73.76	1.26	1.40	0.82	0.60	0.55
32	Brain metastasis	3.99	1.22	1.20	1.19	0.83	0.85	1.99	63.23	1.40	1.52	0.75	0.80	0.44
33	Brain metastasis	1.06	1.41	1.30	1.20	0.76	0.95	1.82	17.51	1.65	1.68	0.70	0.63	0.43
34	Glioblastoma, IDH wildtype	12.11	1.12	1.24	0.52	0.88	0.36	2.63	32.70	1.26	1.30	0.84	0.72	0.51
35	Glioblastoma, IDH wildtype	45.45	1.16	1.36	0.81	0.82	0.44	3.30	89.15	1.31	1.42	0.80	0.74	0.74
36	Glioblastoma, IDH wildtype	41.53	1.59	1.41	1.12	0.69	0.84	2.46	86.06	1.57	1.41	0.70	1.09	0.86
37	Glioblastoma, IDH wildtype	19.01	1.07	1.20	0.71	0.91	0.58	2.06	7.40	1.34	1.39	0.77	0.73	0.72
38	Glioblastoma, IDH wildtype	66.18	1.49	1.58	0.60	0.84	0.52	2.40	173.57	1.59	1.73	0.82	0.55	0.44
39	Glioblastoma, IDH wildtype	5.90	0.91	0.81	1.15	1.00	0.76	1.85	23.00	1.09	1.21	0.93	0.67	0.59
40	Glioblastoma, IDH wildtype	9.25	1.29	1.18	1.26	0.86	1.09	2.07	19.56	1.59	1.63	0.70	0.68	0.57
41	Glioblastoma, IDH wildtype	3.49	0.80	0.79	0.41	1.15	0.06	1.69	26.87	1.35	1.30	0.83	1.11	0.78
42	Glioblastoma, IDH wildtype	79.70	1.47	1.57	0.65	0.82	0.36	2.12	223.01	1.46	1.59	0.80	0.64	0.35
43	Glioblastoma, IDH wildtype	62.90	1.67	1.61	0.79	0.71	0.45	2.12	117.15	1.71	1.65	0.71	0.81	0.47
44	Glioblastoma, IDH wildtype	56.20	1.54	1.65	0.59	0.78	0.40	2.63	129.68	1.56	1.72	0.75	0.55	0.40
45	Glioblastoma, IDH wildtype	28.70	1.56	1.52	0.89	0.76	0.62	2.09	79.37	1.69	1.71	0.68	0.83	0.45
46	Glioblastoma, IDH wildtype	8.50	1.09	1.26	0.69	0.79	0.30	1.92	5.14	1.35	1.83	0.77	0.52	0.25
47	Glioblastoma, IDH wildtype	6.30	1.28	1.70	0.61	0.77	0.30	1.93	4.46	1.25	1.93	0.84	0.51	0.33
48	Glioblastoma, IDH wildtype	4.39	1.41	1.93	0.58	0.68	0.20	1.84	9.59	1.38	1.83	0.72	0.63	0.39
49	Glioblastoma, IDH wildtype	9.96	1.10	1.07	1.32	0.83	0.83	1.93	85.24	1.42	1.39	0.78	0.97	0.72
50	Glioblastoma, IDH wildtype	6.56	1.77	1.51	1.27	0.65	0.51	1.75	257.95	1.57	1.58	0.66	0.88	0.46
51	Glioblastoma, IDH wildtype	11.76	1.26	1.61	0.81	0.84	0.38	2.90	42.82	1.36	1.31	0.81	1.06	0.66
52	Glioblastoma, IDH wildtype	36.40	1.55	1.40	1.09	0.71	0.66	2.73	78.59	1.80	1.78	0.67	0.75	0.40
53	Glioblastoma, IDH wildtype	36.40	1.10	1.12	0.90	0.95	0.86	2.71	30.01	1.46	1.50	0.75	0.62	0.54
54	Glioblastoma, IDH wildtype	24.78	1.25	1.28	0.80	0.89	0.62	1.79	74.17	1.48	1.56	0.80	0.64	0.45
55	Glioblastoma, IDH wildtype	45.70	1.32	1.46	0.65	0.83	0.50	1.93	106.37	1.65	1.90	0.72	0.47	0.30
56	Glioblastoma, IDH wildtype	78.90	0.79	0.62	0.67	0.68	0.50	2.22	95.21	1.30	1.17	0.82	0.86	0.59
57	Glioblastoma, IDH wildtype	7.02	0.99	0.84	0.65	0.61	0.30	2.28	26.30	1.51	1.34	0.77	1.05	0.44
58	Glioblastoma, IDH wildtype	16.70	1.06	0.93	1.10	0.91	1.13	2.31	26.33	1.53	1.45	0.68	0.60	0.63
59	Glioblastoma, IDH wildtype	42.82	1.34	1.41	0.62	0.75	0.47	2.24	75.53	1.67	1.89	0.82	0.52	0.53
60	Glioblastoma, IDH wildtype	21.00	1.07	1.15	0.66	0.95	0.53	1.95	147.42	1.37	1.55	0.83	0.49	0.39
61	Glioblastoma, IDH wildtype	5.49	0.91	0.52	2.08	0.95	1.29	1.92	21.78	1.33	1.18	0.77	1.42	1.82
62	Oligodendroglioma, IDH mutant, WHO 2	1.12	1.05	0.85	2.05	0.95	1.81	1.78	3.75	1.43	1.17	0.82	1.70	1.14
63	Oligodendroglioma, IDH mutant, 1p/19q co‐deleted, WHO 2	2.46	1.56	1.05	1.28	0.57	0.19	2.12	5.99	1.65	1.87	0.77	0.67	0.30
64	Oligodendroglioma, IDH mutant, 1p/19q co‐deleted, WHO 3	0.62	0.84	0.65	2.77	0.91	2.06	2.17	7.03	1.58	1.64	0.74	0.72	0.55
65	Oligodendroglioma, IDH mutant, WHO 3	18.70	0.99	0.78	0.78	0.80	0.40	2.06	48.15	1.36	1.14	0.78	1.00	0.69
66	Oligodendroglioma, IDH mutant, 1p/19q co‐deleted, WHO 3	3.99	1.50	1.75	0.40	0.73	0.45	2.78	44.29	1.46	1.45	0.76	0.85	0.84

**FIGURE 6:**
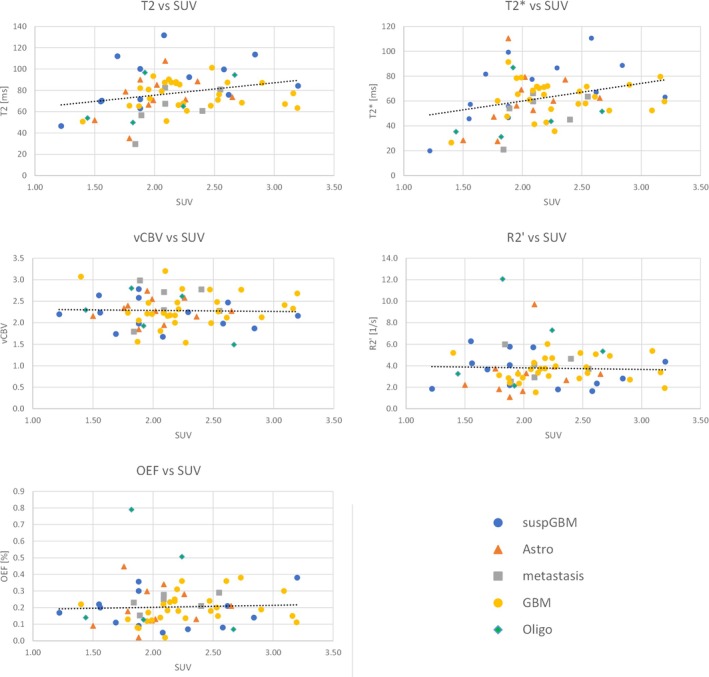
Correlation plot of relative OEF values obtained using GE‐SE EPIK and TBR values from FET PET. The Pearson correlation coefficient is −0.078 with a *p*‐value of 0.53.

The plot of OEF and FET uptake in Figure 6 does not show significant correlation (Pearson correlation coefficient 0.04 with *p*‐value of 0.53). In contrast, a moderate correlation between FET uptake and T2 as well as $T_{2}^{*}$ was found with Pearson correlation coefficients of 0.26 and 0.31, respectively. Both vCBV and $R_{2}^{\prime }$, show no correlation with coefficients of −0.03 and *p*‐values of 0.84 and 0.80.

**FIGURE 7:**
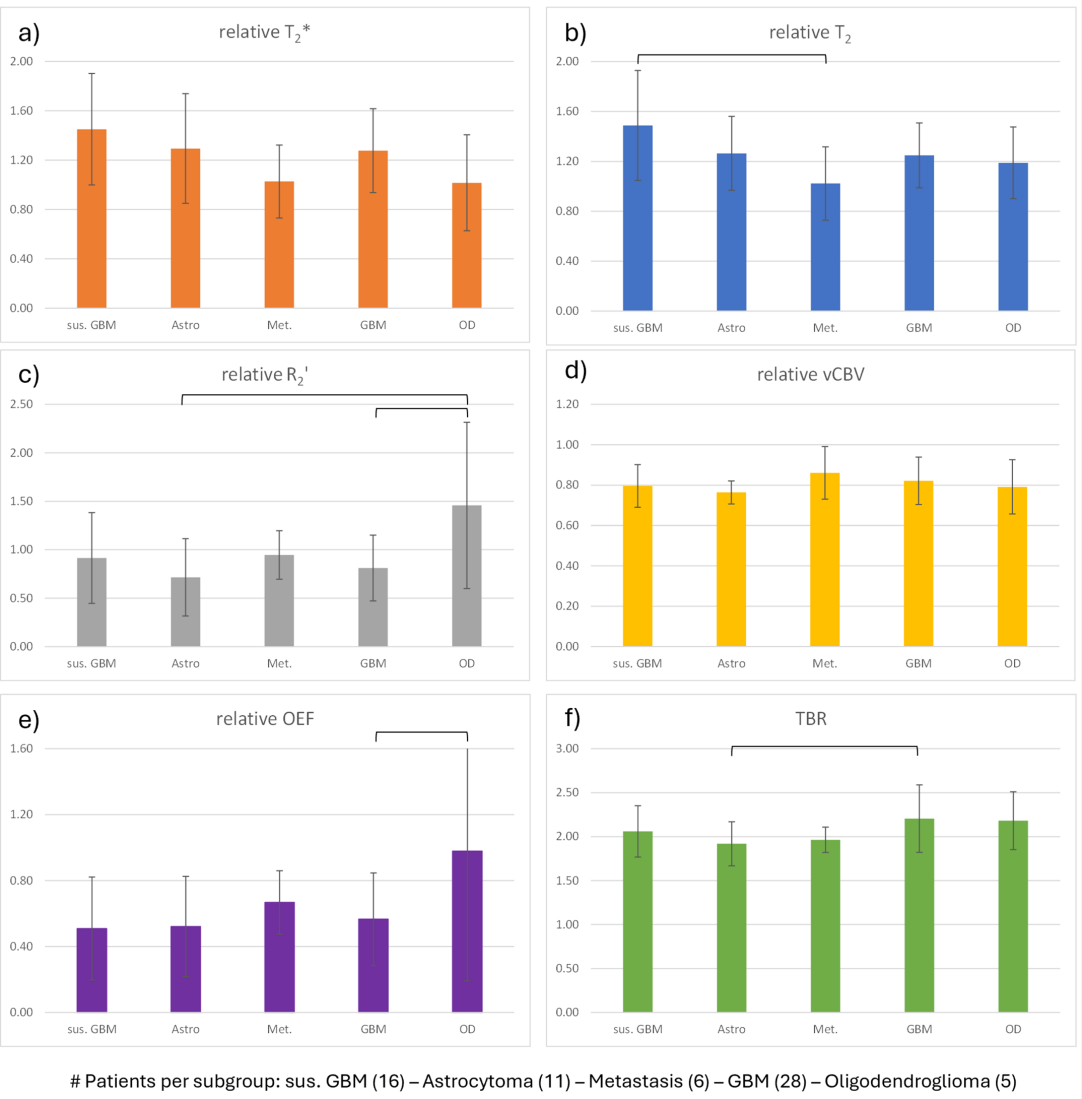
Mean relative measures of quantitative MR parameters (a:T_2_, b: $T_{2}^{*}$, c: $R_{2}^{\prime }$, d:VCBV, e:OEF) and TBRmean (f) evaluated for the subgroups of different tumor types according to the primary histology results. Significant deviations (*p* < 0.05) between subgroups are marked by solid lines, respectively. [sus. GBM = suspected Glioblastoma/Astrocytoma/Metastasis/Glioblastoma/Oligoastrocytoma/Oligodendroglioma].

Figure 7 shows the mean relative values of each quantitative MR parameter from both analyzed VOIs for the different tumor types: untreated suspected glioma (*n* = 16), astrocytoma (*n* = 11), metastasis (*n* = 6), glioblastomas (*n* = 28) and oligodendroglioma (*n* = 5). Oligodendrogliomas had significantly larger ${\mathrm{rR}}_{2}^{\prime }$ compared to glioblastomas and astrocytoma, and significantly larger rOEF compared to glioblastomas. There were significant differences in mean FET TBRs between astrocytomas and glioblastomas. Suspected gliomas were found to have significantly larger T2 compared to astrocytomas. Overall, rvCBV and ${\mathrm{rT}}_{2}^{*}$ showed no significant differences between relative MR measures of different tumor types. A summary table of all derived *p*‐values in this subgroup analysis can be found in the Supporting Information (Table S1).

## 4 Discussion

A previous study [25] successfully implemented and validated the 10‐echo GE‐SE EPIK sequence in 20 healthy subjects, demonstrating strong agreement in relaxation time quantification with reference methods. Additionally, the sensitivity of the quantified OEF values was confirmed during breath‐hold experiments. Building on this, the current study has applied the GE‐SE EPIK sequence to simultaneously quantify transverse relaxation times (T_2_, $T_{2}^{*}$), vCBV, and OEF in brain tumor patients during hybrid MR‐PET acquisitions. The results demonstrate significant changes in all MR parameters, except $R_{2}^{\prime }$, in the tumor VOIs defined by FET PET compared to healthy brain tissue, and the increased transverse relaxation times and reduced OEF in the tumor VOIs are in agreement with previous studies [5, 6, 9, 44]. The resulting MR parameter maps showed additional significant changes in FLAIR‐derived VOIs that extend the tumor VOI defined by FET TBR. In addition, the mean MR parameter values within healthy brain tissue VOIs also agree with those reported in prior studies [25, 39, 42, 45, 46, 47]. The transverse relaxation time parameters, T2 and $T_{2}^{*}$, showed a significant correlation with FET TBR values. However, while the statistical significance of these changes shows the potential of GE‐SE EPIK to identify and characterize tumor tissue in an acquisition time of 2 min for the whole brain, there are limitations that affect its suitability for clinical applications, as discussed in the following paragraphs.

The $T_{2}^{*}$ maps, in particular, contain regions with susceptibility artifacts and hence contain altered values that are hard to distinguish from the changes in tumor tissue. These artifacts further translate into the OEF maps. While these regions appear separated from tumor regions in most patients, additional global effects that extend the tumor VOIs are visible which not only reflect active tumor tissues but also bleeding, inflammation, and treatment‐related changes, as they more closely match the T_2_ hyperintense regions of the FLAIR contrast. All of these influence the quantified relaxation times and hence translate into the assessment of oxygen metabolism. Overall, the resulting OEF method appears noisy, but when used in combination with FET reference VOIs, tumor identification and differentiation become possible. Its value in identifying areas with decreased OEF shows potential, but its sensitivity is limited by proneness to image artifacts and additional tissue alterations. Currently, the MR parameters obtained using GE‐SE EPIK require avisual classification to differentiate pathologic and artifact‐related changes that limit the applicability to automate the analysis of quantified values. To strengthen this identification, the image quality requires improvement by either enhansing SNR by optimizing acquisition parameters, using post‐processing steps to improve contrast‐to‐noise ratio or reducing the strength of the artifacts by employing better shimming. Another future study direction will be to evaluate the capability of parameter combinations to improve tumor tissue identification and tumor type differentiation.

In the current study, the GE‐SE EPIK‐derived OEF values in tumor VOIs were reduced compared to healthy control values, reflecting the expected hypoxia. At the same time, no direct correlation between MR‐rOEF and FET uptake was found. Hence, the reduced OEF in VOIs with increased FET may be associated with other factors, such as hypoxia, ischemia, or anemia.

A shortcoming of using the qBOLD approach to compute OEF based on the signal decay covered by the 10‐echo GE‐SE EPIK sequence is the theoretical distinction between the short‐ and long‐time regimes, which are governed by a quadratic or linear exponential relationship of $R_{2}^{\prime }$, respectively [48]. The critical time separating the regimes depends on the actual OEF value and hence differs for different tissue characteristics. The critical time is measured as the temporal distance from the spin echo (5th echo) and is in the range of the first‐neighbor echoes (4th and 6th), while the second‐neighbor echoes (3rd and 7th) are in the long‐time regime. In contrast to other sequence solutions that sample the decay curve around the SE with a larger number of densely sampled echoes in the range of ΔTE = 3–5 ms (e.g., ASE [49], GESSE [50]), the 10‐echo sequence only covers two data points on each decay tail, and because of this, a linear relationship is assumed. Therefore, a small bias may be present since the next‐neighbor echoes are not fully described by their linear relationship, rather than a transition between both regimes. This limitation effects the vCBV estimation, as it is related to the difference between both regimes. Restrictions in echo sampling may limit the vCBV sensitivity, which is why future studies should consider a separate vCBV acquisition. In addition, the qBOLD theory is based on the static dephasing regime, neglecting any influences from diffusion that may be violated in tumor tissue due to blood–brain‐barrier disruptions.

While the specific investigation of MR parameter changes in well‐defined tumor VOIs showed significant changes compared to contralateral healthy tissues, knowledge of the tumor metabolic tissue boundaries is necessary to accurately distinguish tumor tissue from areas affected by treatment‐related changes or even imaging artifacts. Nevertheless, the results show the potential of the method to reveal the global effects of multiple MR parameters, namely $T_{2}^{*}$, T_2_, vCBV, and OEF, within 2 min of measurement time.

Future work is needed to validate whether the OEF quantification based on GE‐SE EPIK is sensitive to the various tumor characteristics that are shown, for example, in reference‐standard PET acquisitions or whether this kind of OEF model correlates with hypoxia. Hypoxia interpretation has already been reported in the literature; for example, a study on high‐grade glioma patients compared qBOLD‐derived MR‐OEF values with FMISO‐PET [9], one of the most accurate tracers for hypoxia detection. In that study, the authors reported that their comparison did not show a high spatial correspondence. However, in contrast to the methodology in the current study, the MR‐OEF quantification was based on subsequent T_2_ and $T_{2}^{*}$ acquisitions. Further investigations are required to clarify the capabilities of MR‐derived OEF values, for example, by comparison to ^15^O‐labeled radiotracer PET under hypoxic conditions.

For this purpose, these authors aim to perform a comparison study between GE‐SE EPIK‐derived OEF and different MR‐based methods for OEF quantification., such as GESSE or ASE, that can quantify $R_{2}^{\prime }$ as part of the qBOLD methodology. Moreover, comparisons to OEF models based on venous T_2_ quantification, such as those shown by TRUST measurements [51, 52], QSM models [53], or even to the reference‐standard of O^15^ PET measurements under patient‐study conditions will provide valuable insights into the precision and sensitivity of the proposed GE‐SE EPIK model.

However, a noteworthy aspect of the proposed patient study is the potential diversity within a cohort that includes mostly treated patients. Treatment‐related effects play an important role in the interpretability of the data, adding complexity to the tissue heterogeneity. On the one hand, the patient characteristics allowed a direct comparison of different tumor types as well as between a limited number of treated and untreated suspected glioma patients. On the other hand, a more homogeneous study cohort of untreated patients may be beneficial for directly investigating the OEF sensitivity without the influence of treatment‐related changes.

### 4.1 Limitations

This study has several limitations. The number of tumors included was small, particularly for subgroup analysis. The patient cohort was heterogeneous, with varied treatment histories and a limited number of untreated cases. The imaging technique is prone to susceptibility artifacts arising from air‐tissue interfaces. Additionally, the sensitivity of vCBV quantification was limited due to restrictions in the echo timing and sampling within the static dephasing regime.

### 4.2 Conclusions

Compared to healthy reference regions, the tumor regions showed a significant increase in T_2_ and $T_{2}^{*}$, while vCBV and OEF were significantly reduced, agreeing with the expected hypoxic tissue behavior. There was no correlation between FET uptake and MR‐derived parameters, showing that the MR‐derived parameters may potentially provide independent and added value compared to PET values. Some significant differences were found between tumor types in $T_{2}^{*}$, $R_{2}^{\prime }$, and OEF. Treatment‐related changes and imaging artifacts resulted in global alterations in MR parameters that extend beyond the tumor VOIs. This limits the ability of the GE‐SE EPIK sequence to identify tumor tissue characteristics and distinguish recurrent tumor tissue from treatment‐related changes.

## Supporting information


**Figure S1.** Representative images of the anatomical T1 MP‐RAGE scan (top), T2w FLAIR images (2nd row), the FET SUV map (3rd row) and OEF maps (bottom) for different tumor types, that is, astrocytoma, glioblastoma, metastasis, and oligodendroglioma, from left to right. Tumor VOIs derived from FET PET thresholds are overlayed with pink outlines and FLAIR‐derived VOIs in orange.

**Figure S2.** Representative images of the quantitative parameter maps from the 10‐echo GE‐SE EPIK sequence. From top to bottom, T_2_*, T_2_, R_2_^′^ and vCBV are shown for different tumor types, that is, astrocytoma, glioblastoma, metastasis, and oligodendroglioma, from left to right. Tumor VOIs derived from FET PET thresholds are overlayed with pink outlines and FLAIR‐derived VOIs in orange.

**Table S1.** Summary of *p*‐values for the tumor type differentiation analysis based on FET‐derived VOIs (a) and FLAIR derived VOIs (b). *p*‐values from two‐tailed *t*‐tests are given for each compared tumor type subgroup combination and for each MR‐parameter.

## Data Availability

The original in vivo data can be shared by submitting a request to the corresponding author (N. Jon Shah: n.j.shah@fz-juelich.de) under a formal data‐sharing agreement.
