# Supplementary figures and images for: Brain Tumor Characterization Using Multiple MR Parameters From Multi‐Contrast EPI With Keyhole (GE‐SE EPIK) Including Oxygen Extraction Fraction: A Comparison to O‐(2‐[18F]Fluoroethyl)‐L‐Tyrosine (FET) Positron Emission Tomography
Source: J Magn Reson Imaging. 2025 Apr 17;62(3):721–36. doi: 10.1002/jmri.29795 (PMC12335343; doi:10.1002/jmri.29795)

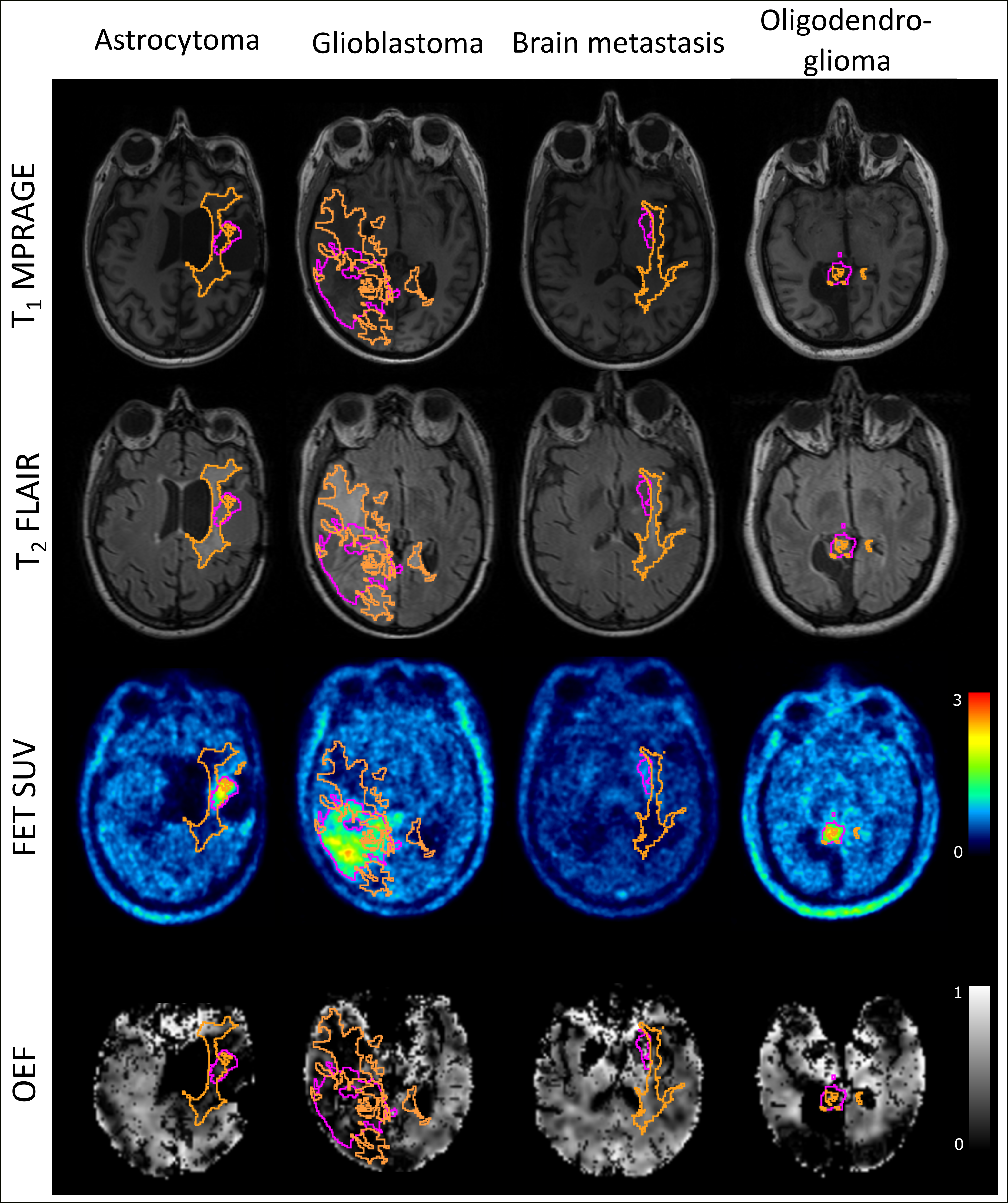

Supplement: Supplementary file 1 — Figure S1. Representative images of the anatomical T1 MP‐RAGE scan (top), T2w FLAIR images (2nd row), the FET SUV map (3rd row) and OEF maps (bottom) for different tumor types, that is, astrocytoma, glioblastoma, metastasis, and oligodendroglioma, from left to right. Tumor VOIs derived from FET PET thresholds are overlayed with pink outlines and FLAIR‐derived VOIs in orange. [file JMRI-62-721-s002.tiff]

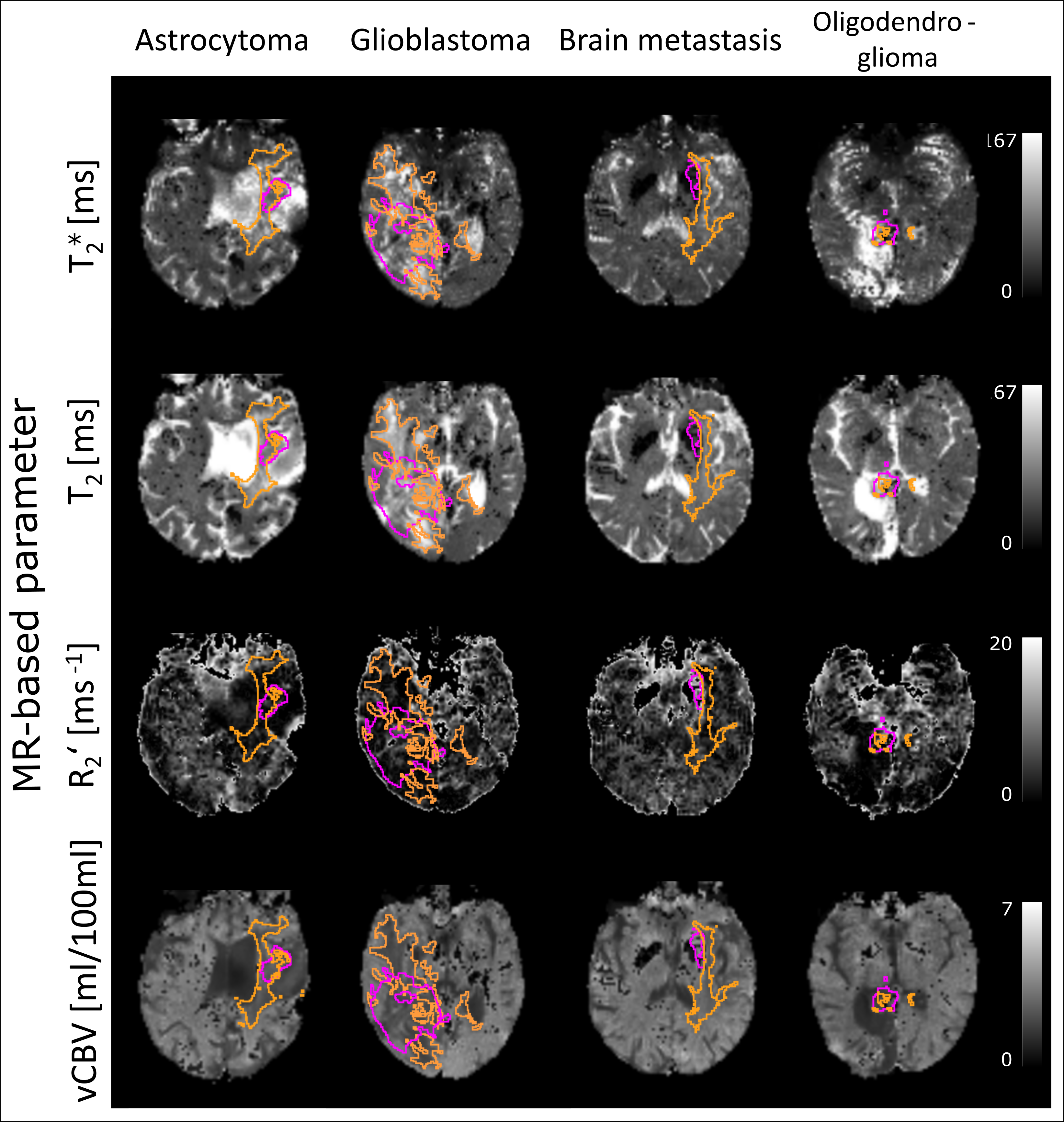

Supplement: Supplementary file 2 — Figure S2. Representative images of the quantitative parameter maps from the 10‐echo GE‐SE EPIK sequence. From top to bottom, T2*, T2, R2 ′ and vCBV are shown for different tumor types, that is, astrocytoma, glioblastoma, metastasis, and oligodendroglioma, from left to right. Tumor VOIs derived from FET PET thresholds are overlayed with pink outlines and FLAIR‐derived VOIs in orange. [file JMRI-62-721-s003.tiff]
