# Supplementary material for: Brain Tumor Characterization Using Multiple MR Parameters From Multi‐Contrast EPI With Keyhole (GE‐SE EPIK) Including Oxygen Extraction Fraction: A Comparison to O‐(2‐[18F]Fluoroethyl)‐L‐Tyrosine (FET) Positron Emission Tomography
Source: J Magn Reson Imaging. 2025 Apr 17;62(3):721–36. doi: 10.1002/jmri.29795 (PMC12335343; doi:10.1002/jmri.29795)
Supplement: Supplementary file 3 — Table S1. Summary of p‐values for the tumor type differentiation analysis based on FET‐derived VOIs (a) and FLAIR derived VOIs (b). p‐values from two‐tailed t‐tests are given for each compared tumor type subgroup combination and for each MR‐parameter. [file JMRI-62-721-s001.docx]

**Supplementary Table S1**

| (a) FET- SUV VOIs | Typ1 Typ2 | sus. GBM  Astro | sus. GBM  Met. | sus. GBM  GBM | sus. GBM  OD | Astro  Met. | Astro  GBM | Astro  OD | Met.  GBM | Met.  OD | GBM  OD |
| --- | --- | --- | --- | --- | --- | --- | --- | --- | --- | --- | --- |
|  | T_2_ | 0.168 | 0.033 | 0.080 | 0.190 | 0.151 | 0.877 | 0.661 | 0.077 | 0.417 | 0.652 |
|  | T_2_* | 0.415 | 0.066 | 0.422 | 0.094 | 0.235 | 0.899 | 0.280 | 0.116 | 0.964 | 0.146 |
|  | R_2_’ | 0.276 | 0.779 | 0.645 | 0.101 | 0.136 | 0.285 | 0.044 | 0.300 | 0.305 | 0.013 |
|  | vCBV | 0.418 | 0.124 | 0.193 | 0.673 | 0.205 | 0.504 | 0.931 | 0.489 | 0.456 | 0.620 |
|  | OEF | 0.965 | 0.280 | 0.549 | 0.079 | 0.332 | 0.673 | 0.139 | 0.419 | 0.418 | 0.048 |
|  | TBR | 0.220 | 0.471 | 0.205 | 0.464 | 0.706 | 0.031 | 0.124 | 0.150 | 0.221 | 0.897 |
| (b) FLAIR VOIs | Typ1 Typ2 | sus. GBM  Astro | sus. GBM  Met. | sus. GBM  GBM | sus. GBM  OD | Astro  Met. | Astro  GBM | Astro  OD | Met.  GBM | Met.  OD | GBM  OD |
|  | T_2_ | 0.167 | 0.257 | 0.063 | 0.464 | 0.999 | 0.905 | 0.660 | 0.921 | 0.625 | 0.658 |
|  | T_2_* | 0.437 | 0.962 | 0.749 | 0.478 | 0.480 | 0.438 | 0.891 | 0.831 | 0.435 | 0.445 |
|  | R_2_’ | 0.954 | 0.096 | 0.092 | 0.727 | 0.073 | 0.082 | 0.758 | 0.223 | 0.076 | 0.074 |
|  | vCBV | 0.123 | 0.296 | 0.072 | 0.228 | 0.743 | 0.884 | 0.969 | 0.828 | 0.722 | 0.943 |
|  | OEF | 0.757 | 0.206 | 0.522 | 0.701 | 0.115 | 0.781 | 0.431 | 0.275 | 0.069 | 0.336 |

Table S1: Summary of p-values for the tumor type differentiation analysis based on FET-derived VOIs (a) and FLAIR derived VOIs (b). P-values from two-tailed t-tests are given for each compared tumor type subgroup combination and for each MR-parameter.

Supplementary Figure 1

*Supplementary Figure 1: Representative images of the anatomical T1 MP-RAGE scan (top), T2w Flair images (2^nd^ row), the FET SUV map (3^rd^ row) and OEF maps (bottom) for different tumor types, i.e. astrocytoma, glioblastoma, metastasis, and oligodendroglioma, from left to right. Tumor VOIs derived from FET PET thresholds are overlayed with pink outlines and FLAIR-derived VOIs in orange.*

Supplementary Figure 2

*Supplementary Figure 2: Representative images of the quantitative parameter maps from the 10-echo GE-SE EPIK sequence. From top to bottom, T_2_*, T_2_, R_2_’ and vCBV are shown for different tumor types, i.e. astrocytoma, glioblastoma, metastasis, and oligodendroglioma, from left to right. Tumor VOIs derived from FET PET thresholds  are overlayed with pink outlines and FLAIR-derived VOIs in orange.*
